# Endoscopic Stenting in Hilar Cholangiocarcinoma: When, How, and How Much to Drain?

**DOI:** 10.1155/2019/5161350

**Published:** 2019-11-04

**Authors:** Andrea Tringali, Ivo Boškoski, Guido Costamagna

**Affiliations:** ^1^Digestive Endoscopy Unit, Fondazione Policlinico Universitario A. Gemelli IRCCS, Rome, Italy; ^2^Centre for Endoscopic Research Therapeutics and Training (CERTT), Università Cattolica del Sacro Cuore, Rome, Italy

## Abstract

Hilar cholangiocarcinoma (HCCA) involves a complex anatomical region where bile ducts, arteries, and veins create a complex network. HCCA can lead to biliary strictures at the main hepatic confluence, involving the right and left radicles. Endoscopic drainage of jaundiced patients with HCCA is challenging and carries a high risk of infective complications. HCCA needs a careful multidisciplinary evaluation to assess the indication and purposes (preoperative/palliative) of the biliary drainage. Biliary drainage in HCCA needs to be planned by magnetic resonance cholangiography in order to study the biliary anatomy and perform a target drainage of the intrahepatic ducts above the malignant hilar stricture; all the opacified intrahepatic ducts above the hilar stricture must be drained to reduce septic complications. Drainage of >50% of the liver volume is important to obtain bilirubin reduction and less complications, but atrophic liver segments (identified by CT scan) do not require drainage due to the increased risk of cholangitis. When preoperative biliary drainage is planned, plastic stents must be inserted. Self-expandable metal stents are indicated for palliative purposes and should be placed only when a complete liver drainage is possible; only uncovered metal stents are indicated to drain malignant hilar strictures to avoid side-branch occlusion.

## 1. Introduction

Endoscopic drainage of hilar cholangiocarcinoma (HCCA) is a technically demanding procedure due to the tumor location which can obstruct several intrahepatic radicles at the main hepatic confluence. Endoscopic and percutaneous biliary drainages are the available techniques to treat jaundice secondary to HCCA. ERCP provides internal drainage by insertion of multiple plastic or metal stents, with a better effect on the quality of life compared to percutaneous drains [1, 2]. Both techniques resulted in effective jaundice resolution, and recent guidelines propose their use to be modulated according to the local expertise [3]. EUS-guided transgastric drainage is another available technique to drain HCCA but data and results are still limited.

Infectious complications are the “Achilles' heel” of both the endoscopic and percutaneous drainages of HCCA due to the contamination of the intrahepatic ducts above the complex malignant hilar stricture (MHS). Suboptimal drainage of intrahepatic ducts is the main reason for the high rate of cholangitis secondary to MHS drainage [4].

Magnetic resonance cholangiography (MRC) can provide a detailed “road-map” to perform optimal biliary drainage of HCCA, thus reducing the rate of infective complications avoiding the scenario of “opacified and undrained biliary ducts.”

## 2. Anatomical Considerations

When approaching MHS secondary to HCCA, the normal anatomy of the intrahepatic ducts should be considered to obtain effective drainage.

**Figure 1:**
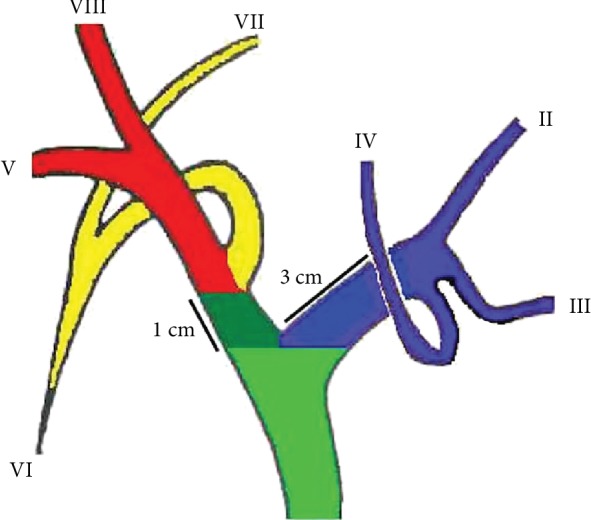
Bile duct anatomy. The left hepatic duct is 3 cm long. The right hepatic duct (dark green) is 1 cm long before dividing into the right anterior (red) and right posterior (yellow) sectorial ducts.

The left hepatic duct is usually 3 cm long before dividing into the ducts for segments 2, 3, and 4, while the right hepatic duct is about 1 cm in length and divides early into the two sectorial ducts (anterior for segments 5 and 8, posterior for segments 6 and 7) [5] (Figure 1). Thus, as HCCA advances, right lobe sectors are involved and obstructed earlier compared to the left. The two right sectorial ducts (anterior and posterior), when separated, require 2 stents to obtain complete drainage of the right liver lobe. Segment 1 has its own biliary drainage into both hepatic ducts and its biliary ducts are rarely injected during cholangiography.

## 3. Classification of Malignant Hilar Biliary Strictures

The degree of involvement of the intrahepatic ducts in HCCA is commonly classified according to the Bismuth classification [6, 7]. This is a surgical classification which should drive the operative strategy (tumor local excision with or without extended liver resections). Nevertheless, the Bismuth classification is widely used by radiologists and endoscopists because it describes the extent of the tumor into the intrahepatic ducts, which can have a practical impact on drainage strategy.

*Type I*: only the proximal common hepatic duct is involved, without reaching the main hepatic confluence; it is a nonhilar stricture, 1 stent/PTBD can drain the whole liver.

**Figure 2:**
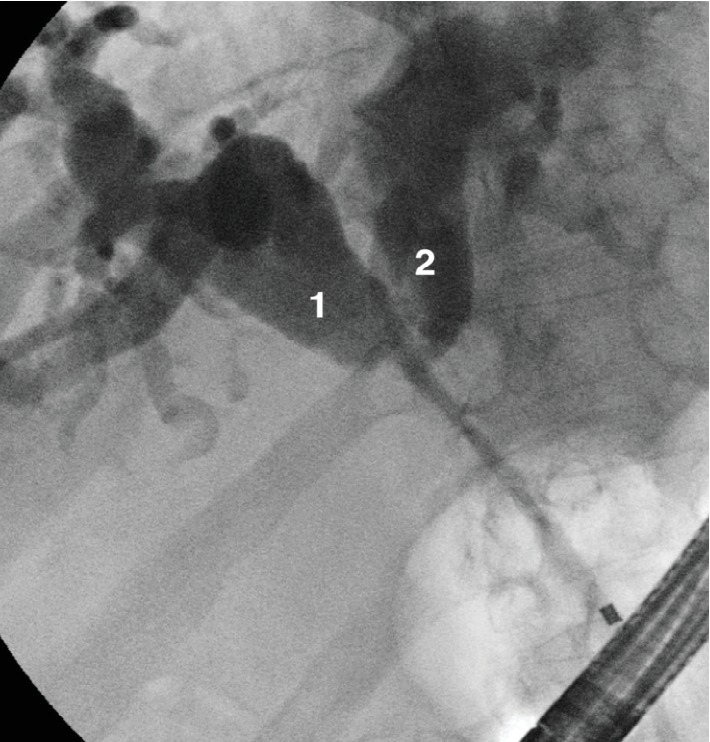
Bismuth type II hilar stricture: the main hepatic confluence is separated; 2 stents are needed to drain the whole liver.

*Type II*: the main hepatic confluence is separated; 2 stents/PTBDs are needed to obtain complete liver drainage (Figure 2).

**Figure 3:**
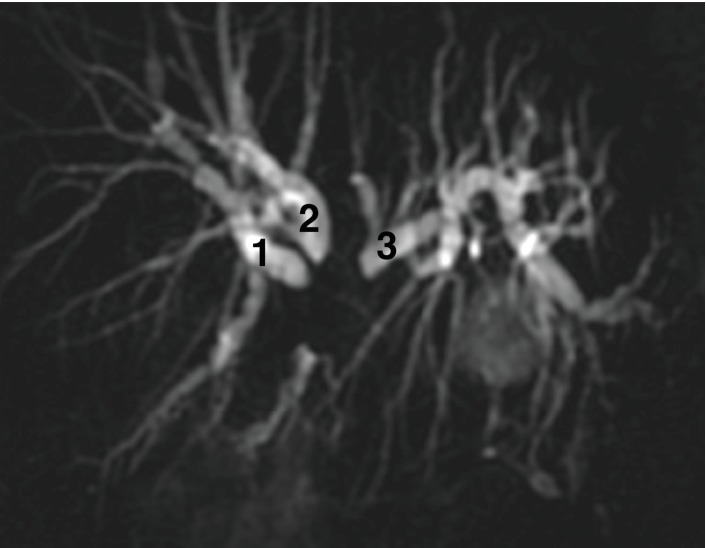
Bismuth type IIIa hilar stricture: the main hepatic confluence and the right secondary are involved; 3 stents are needed to drain the whole liver.

*Types IIIa and IIIb*: the main hepatic confluence and the right (anterior/posterior) or left (IV/II and III segments) secondary branches are obstructed; 3 stents/PTBDs can drain all the intrahepatic ducts (Figure 3).

*Type IV*: reflects an advanced disease with involvement of both the primary and secondary right and left biliary confluences; theoretically, 4 stents/PTBDs are needed.

**Figure 4:**
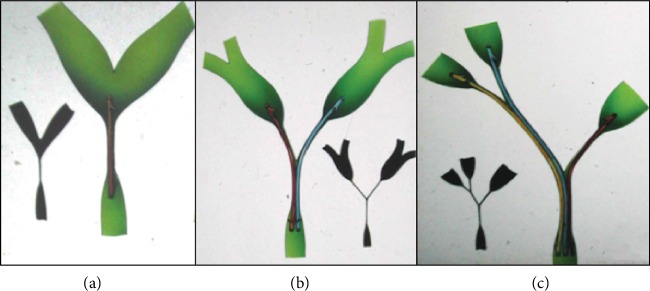
Schematic approach to complete endoscopic drainage in Bismuth type I ((a) one stent), II ((b) two stents), and IIIa ((c) three stents) malignant hilar strictures.

A schematic approach to stenting in MHS according to Bismuth classification is summarized in Figure 4.

## 4. Biliary Drainage in Malignant Hilar Strictures

### 4.1. Unilateral/Bilateral

Published studies simplify MHS drainage into two strategies: *unilateral* and *bilateral* depending on the number of inserted stents (one = unilateral, two = bilateral) [8, 9]. These definitions apply to the Bismuth type II strictures (Figure 2) where the right and left biliary systems are separated at the main hepatic confluence; insertion of one stent is enough to obtain *unilateral* (right or left liver lobe) biliary drainage, while two stents are needed to achieve *bilateral* drainage including the whole liver.

**Figure 5:**
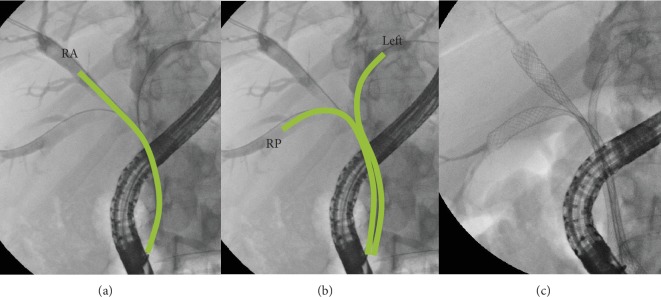
Bismuth type IIIa hilar stricture. One (a) or 2 (b) stents obtain an incomplete liver drainage; 3 stents are needed for a complete liver drainage (c). RA: right anterior; RP: right posterior.

When the MHS is more complex (i.e., Bismuth IIIa), one stent can obtain partial liver drainage (left, right anterior or right or posterior) (Figure 5(a)), and also two stents are not enough to drain the whole liver (Figure 5(b)): three stents are needed to obtain complete liver drainage (Figure 5(c)).

### 4.2. Complete/Incomplete

Due to MHS complexity, draining all the intrahepatic ducts is not always technically feasible. The terms *complete* (all intrahepatic ducts) and *incomplete* (one or some intrahepatic duct) biliary drainage reflect the amount of liver parenchyma that is successfully drained. These definitions seem more appropriate than unilateral and bilateral, which cannot be applied to all types of MHS.

### 4.3. How Much to Drain?

Historically, it was assumed that drainage of about 25-30% (i.e., the left biliary system alone) of the liver parenchyma could be enough to obtain relief of obstructive jaundice [10, 11].

Hintze et al. [12] introduced magnetic resonance cholangiography (MRC) as a diagnostic tool to identify the best liver segment to drain during ERCP in 35 nonresectable Klatskin tumors (13 Bismuth type III and 22 type IV). Cannulation of the MRC-selected duct to drain was achieved under fluoroscopic control with a catheter and guidewire, and then contrast was injected proximally to the stricture avoiding opacification of other ducts. A single 10 Fr plastic stent was inserted in the opacified duct with jaundice resolution in 86% of the cases. The rate of cholangitis during the first 30 days was 6%. This single arm study described promising results when performing *incomplete* drainage of MHS but was limited by the absence of a control group.

A French study [13] analyzed, by CT scan, liver volumetry of the three main liver segments (left, right anterior, and right posterior) in 107 patients that underwent endoscopic stenting of MHS (Bismuth type ≥ 2); drained liver volume was classified into 3 groups: less than 30%, 30% to 50%, and more than 50%. These authors concluded that stenting more than 50% of the liver volume is an independent factor significantly contributing to a greater decrease in bilirubin level, a lower incidence of early cholangitis, and longer survival. Similar results were reported in other two studies [14, 15].

When approaching MHS, recent guidelines [3] recommend to *drain* ≥ 50%*of the liver volume* and to avoid the opacification of biliary ducts that will not be drained.

### 4.4. Which Liver Parenchyma Should be Drained?

Evaluation of the “quality” of the liver parenchyma before draining HCCA is another important issue. In the setting of HCCA, monolateral portal vein thrombosis and subsequent segmental liver atrophy can occur. CT scan/MRI is essential to study liver parenchymal atrophy.

Drainage of an atrophic liver segment should be avoided because clinical success and survival are not increased [16], and the risk of cholangitis is even significantly higher [13].

## 5. Endoscopic Stenting in Hilar Cholangiocarcinoma

### 5.1. When Is Stenting Needed?

Jaundice is the typical clinical manifestation of HCCA. Immediate biliary drainage should be avoided because stents or percutaneous drains impair tumor staging by cross-sectional imaging modalities (CT scan, MRI/MRC), due to image artifacts [3, 17]. MRC is also important to plan the biliary drainage strategy, and therefore can be considered a prerequisite before any attempt to biliary drainage [3, 12].

The aim of biliary drainage in HCCA should be decided in the setting of a multidisciplinary team [3]. Surgery is the only curative option for HCCA and improper biliary drainage, leading to infective complications, can transform a potentially resectable patient into a nonoperable one.

If the patient is a candidate to left hepatectomy, preoperative biliary drainage is not indicated [18, 19], while it is required when estimated future liver remnant volume is <30% and portal vein embolization is needed to obtain hypertrophy of the remnant liver after surgery [20, 21].

Patients that are not a candidates to surgery receive jaundice palliation by stent insertion and histological characterization for further radiochemotherapy. ERCP remains the method of choice to perform brush cytology and/or intraductal biopsy. Recent advances in cholangioscopy made direct inspection with target biopsy of MHS [22, 23] reproducible and widely available in clinical practice. As far as EUS is concerned, the experience with staging and sampling of MHS is still limited [3].

### 5.2. Plastic Stents

Plastic stenting is commonly the first-line drainage method for jaundice relief in HCCA. Some characteristics of plastic stents (PS) should be considered:

- Removability
- The insertion does not impair the subsequent therapeutic plan
- Other PS can be added in case of ineffective biliary drainage
- PS size can be adapted to the common bile duct diameter which is not dilated in HCCA

PS are indicated for preoperative biliary drainage and when no final decision about curative/palliative treatment has been taken.

**Figure 6:**
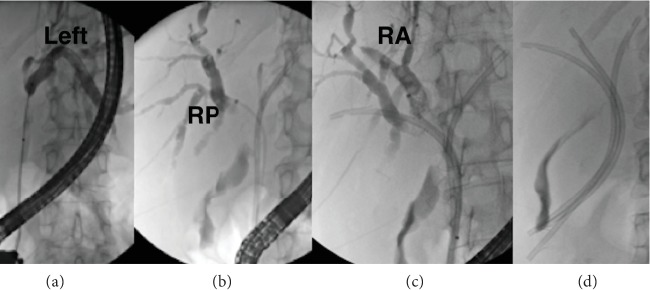
Bismuth type IIIA hilar stricture. Selective cannulation with antegrade opacification of the left (a), right posterior (b), and right anterior (c) biliary ducts obtaining a complete liver drainage (d). RA: right anterior; RP: right posterior.

**Figure 7:**
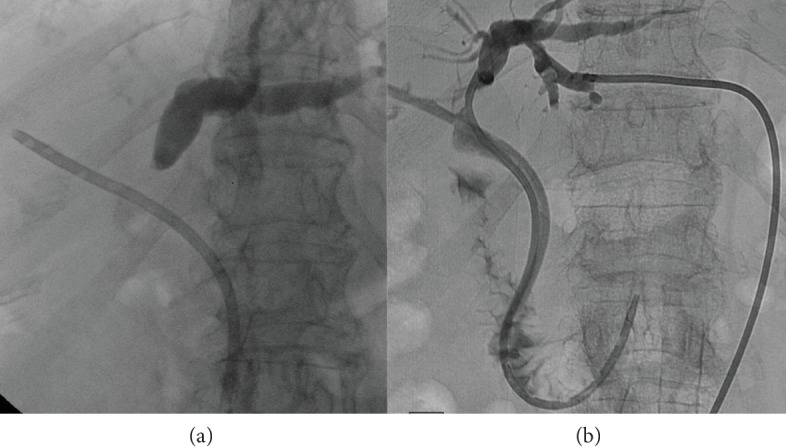
Opacified and undrained left hepatic duct in hilar cholangiocarcinoma (a). Percutaneous drainage obtained a complete liver drainage (b).

Insertion of multiple plastic stents in HCCA should be performed by (1) selective cannulation with a guidewire of the intrahepatic ducts above the hilar stricture; (2) antegrade contrast injection to define the anatomy and perform a selective drainage of the biliary duct; (3) balloon dilatation (4-6 mm) of the stricture, if needed; (4) plastic stent insertion with drainage of the opacified duct, thus avoiding the scenario of “injected and undrained ducts” (Figure 6); plastic stents' length needs to be tailored case by case, but 12 cm long stents are more frequently used. In the case of opacified and undrained biliary ducts, percutaneous drainage should be performed as soon as possible [3] (Figure 7). To reduce the risk of cholangitis, antegrade injection of air, instead of contrast, was proposed, but the risk of infection does not seem significantly reduced [3].

Japanese authors proposed preoperative biliary drainage of the future remnant liver by nasobiliary drains with reintegration of the same bile by mouth or through a nasogastric tube; results of this kind of treatment seem promising but further data are expected [24].

### 5.3. Uncovered Self-Expandable Metal Stents (U-SEMS)

Among the various types of SEMS (uncovered, partially covered, fully covered), only U-SEMS are recommended in MHS because the drainage of the side branches is possible through the uncovered meshes [3, 25]. U-SEMS are not removable, and their presence at the hepatic hilum can preclude surgery; for these reasons, U-SEMS are indicated only for *palliative* purposes in HCCA [3].

**Figure 8:**
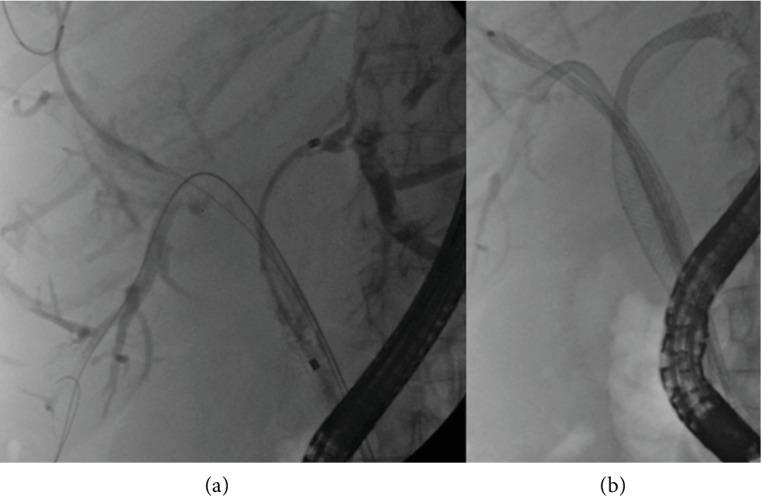
Bismuth type IIIa hilar stricture (a). Three uncovered self-expandable metal stents are inserted side-by-side for palliative purposes (b).

Insertion of multiple U-SEMS “side-by-side” is easier when plastic stents, obtaining complete biliary drainage, have been previously placed; placement of U-SEMS in HCCA should be performed only when all the intrahepatic ducts have been properly cannulated (Figure 8); usually 8 or 10 (rarely 12) cm long U-SEMS are needed to drain MHS.

**Figure 9:**
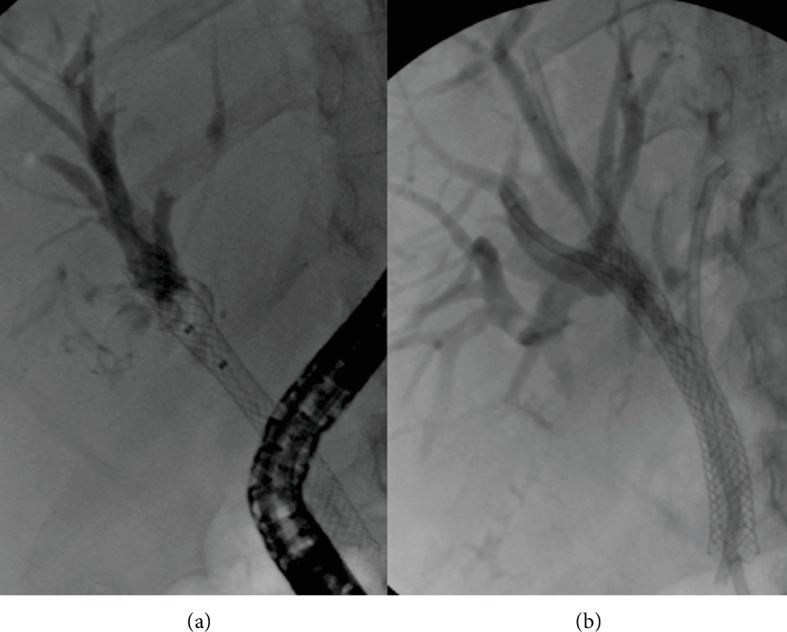
Complex hilar stricture. A single uncovered self-expandable metal stent has been inserted into the right anterior duct obstructing the other intrahepatic ducts (a), leading to cholangitis. Left and right posterior ducts are drained by plastic stents through the meshes (b).

The importance of complete biliary drainage in HCCA is underlined by the difficulties in draining intrahepatic ducts through the meshes of an improperly placed U-SEMS (Figure 9).

As previously discussed, incomplete biliary drainage in HCCA can be considered in the presence of segmental atrophy.

U-SEMS are more expensive than plastic, but their patency is significantly longer thus reducing the need for further hospitalization due to cholangitis and stent malfunction [9, 26].

U-SEMS in HCCA can be inserted “side-by-side” or “stent-in-stent”; the latter configuration is complex, placement of more than 2 stents is rarely reported, and retreatment is challenging and not always possible [27, 28]. For these reasons, “side-by-side” multiple U-SEMS is the preferred configuration for HCCA palliation because retreatments are easy and successful, especially if the U-SEMS are placed transpapillary [29]. The possibility to retreat malfunctioning multiple “side-by-side” SEMS seem important because survival resulted significantly higher in patients who received retreatments, according to a large series on 134 patients [29].

U-SEMS can occlude because of sludge deposition or tissue ingrowth; sludge can be removed with a Fogarty balloon, while ingrowth can be resolved by insertion of a second U-SEMS or a plastic stent according to life expectancy.

SEMS patency can be increased by adding photodynamic therapy or radiofrequency ablation before stent insertion [30, 31]. These techniques seem promising but are not widely diffuse; data are limited and cost-effective analysis is lacking.

## 6. Conclusions

Endoscopic drainage of HCCA is challenging and its approach should be performed in referral centers where a multidisciplinary team (radiologist, interventional radiologist, surgeon, endoscopist, pathologist) [3] is available.

The first step is staging HCCA and assessing its surgical resectability; at this point, the aim of the biliary drainage can be established.

- *Preoperative biliary drainage* is indicated in case of extended right hepatectomy performing complete drainage of the future remnant liver before portal vein embolization; in case of left hepatectomy, biliary drainage is not routinely indicated, if necessary, the future remnant liver should be drained. Preoperative drainage is performed with *plastic* stents only
- *Palliative biliary drainage* can be done by insertion of multiple plastic stents or U-SEMS

Among SEMS design, only *uncovered* stents are indicated for HCCA palliation to avoid occlusion of side branches. It is suggested to place multiple U-SEMS side-by-side and transpapillary to facilitate retreatments.

Complete drainage of all the intrahepatic is desired to reduce infective complications; atrophic liver parenchyma does not need biliary drainage. Opacified biliary ducts above MHS must be drained to reduce infective complications.

## References

[1] Luman W., Cull A., Palmer K. R. (1997). Quality of life in patients stented for malignant biliary obstructions. *European Journal of Gastroenterology & Hepatology*.

[2] Born P., Rösch T., Triptrap A. (1998). Long-term results of percutaneous transhepatic biliary drainage for benign and malignant bile duct strictures. *Scandinavian Journal of Gastroenterology*.

[3] Dumonceau J. M., Tringali A., Papanikolaou I. S. (2018). Endoscopic biliary stenting: indications, choice of stents, and results: European Society of Gastrointestinal Endoscopy (ESGE) Clinical Guideline—updated October 2017. *Endoscopy*.

[4] Moole H., Dharmapuri S., Duvvuri A. (2016). Endoscopic versus percutaneous biliary drainage in palliation of advanced malignant hilar obstruction: a meta-analysis and systematic review. *Canadian Journal of Gastroenterology & Hepatology*.

[5] Tibble J. A., Cairns S. R. (2001). Role of endoscopic endoprostheses in proximal malignant biliary obstruction. *Journal of Hepato-Biliary-Pancreatic Surgery*.

[6] Bismuth H., Corlette M. B. (1975). Intrahepatic cholangioenteric anastomosis in carcinoma of the hilus of the liver. *Surgery, Gynecology & Obstetrics*.

[7] Bismuth H., Nakache R., Diamond T. (1992). Management strategies in resection for hilar cholangiocarcinoma. *Annals of Surgery*.

[8] Hong W., Sun X., Zhu Q. (2013). Endoscopic stenting for malignant hilar biliary obstruction: should it be metal or plastic and unilateral or bilateral?. *European Journal of Gastroenterology & Hepatology*.

[9] Sawas T., Al Halabi S., Parsi M. A., Vargo J. J. (2015). Self-expandable metal stents versus plastic stents for malignant biliary obstruction: a meta-analysis. *Gastrointestinal Endoscopy*.

[10] Dowsett J. F., Vaira D., Hatfield A. R. W. (1989). Endoscopic biliary therapy using the combined percutaneous and endoscopic technique. *Gastroenterology*.

[11] Baer H. U., Rhyner M., Stain S. C. (1994). The effect of communication between the right and left liver on the outcome of surgical drainage for jaundice due to malignant obstruction at the hilus of the liver. *HPB Surgery*.

[12] Hintze R. E., Abou-Rebyeh H., Adler A., Veltzke-Schlieker W., Felix R., Wiedenmann B. (2001). Magnetic resonance cholangiopancreatography-guided unilateral endoscopic stent placement for Klatskin tumors. *Gastrointestinal Endoscopy*.

[13] Vienne A., Hobeika E., Gouya H. (2010). Prediction of drainage effectiveness during endoscopic stenting of malignant hilar strictures: the role of liver volume assessment. *Gastrointestinal Endoscopy*.

[14] Takahashi E., Fukasawa M., Sato T. (2015). Biliary drainage strategy of unresectable malignant hilar strictures by computed tomography volumetry. *World Journal of Gastroenterology*.

[15] Bulajic M., Panic N., Radunovic M. (2012). Clinical outcome in patients with hilar malignant strictures type II Bismuth-Corlette treated by minimally invasive unilateral versus bilateral endoscopic biliary drainage. *Hepatobiliary & Pancreatic Diseases International*.

[16] Gwon D. I., Ko G. Y., Sung K. B. (2011). Percutaneous biliary metallic stent placement in patients with unilobar portal vein occlusion caused by advanced hilar malignancy: outcome of unilateral versus bilateral stenting. *American Journal of Roentgenology*.

[17] Mansour J. C., Aloia T. A., Crane C. H., Heimbach J. K., Nagino M., Vauthey J. N. (2015). Hilar cholangiocarcinoma: expert consensus statement. *HPB*.

[18] Liu F., Li Y., Wei Y., Li B. (2011). Preoperative biliary drainage before resection for hilar cholangiocarcinoma: whether or not? A systematic review. *Digestive Diseases and Sciences*.

[19] Celotti A., Solaini L., Montori G., Coccolini F., Tognali D., Baiocchi G. (2017). Preoperative biliary drainage in hilar cholangiocarcinoma: systematic review and meta-analysis. *European Journal of Surgical Oncology*.

[20] Wiggers J. K., Groot Koerkamp B., Cieslak K. P. (2016). Postoperative mortality after liver resection for perihilar cholangiocarcinoma: development of a risk score and importance of biliary drainage of the future liver remnant. *Journal of the American College of Surgeons*.

[21] Olthof P. B., Wiggers J. K., Groot Koerkamp B. (2017). Postoperative Liver Failure Risk Score: Identifying Patients with Resectable Perihilar Cholangiocarcinoma Who Can Benefit from Portal Vein Embolization. *Journal of the American College of Surgeons*.

[22] Navaneethan U., Hasan M. K., Kommaraju K. (2016). Digital, single-operator cholangiopancreatoscopy in the diagnosis and management of pancreatobiliary disorders: a multicenter clinical experience (with video). *Gastrointestinal Endoscopy*.

[23] Badshah M. B., Vanar V., Kandula M. (2019). Peroral cholangioscopy with cholangioscopy-directed biopsies in the diagnosis of biliary malignancies: a systemic review and meta-analysis. *European Journal of Gastroenterology & Hepatology*.

[24] Kawashima H., Itoh A., Ohno E. (2013). Preoperative endoscopic nasobiliary drainage in 164 consecutive patients with suspected perihilar cholangiocarcinoma: a retrospective study of efficacy and risk factors related to complications. *Annals of Surgery*.

[25] Costamagna G., Tringali A. (2017). Can we insert a covered stent, partially or not, in case of hilar biliary stenosis?. *Endoscopy International Open*.

[26] Zorrón Pu L., de Moura E. G., Bernardo W. M. (2015). Endoscopic stenting for inoperable malignant biliary obstruction: a systematic review and meta-analysis. *World Journal of Gastroenterology*.

[27] Lee T. H., Moon J. H., Kim J. H. (2013). Primary and revision efficacy of cross-wired metallic stents for endoscopic bilateral stent-in-stent placement in malignant hilar biliary strictures. *Endoscopy*.

[28] Lee T. H., Park D. H., Lee S. S. (2013). Technical feasibility and revision efficacy of the sequential deployment of endoscopic bilateral side-by-side metal stents for malignant hilar biliary strictures: a multicenter prospective study. *Digestive Diseases and Sciences*.

[29] Boškoski I., Tringali A., Familiari P. (2019). A 17 years retrospective study on multiple metal stents for complex malignant hilar biliary strictures: Survival, stents patency and outcomes of re- interventions for occluded metal stents. *Digestive and Liver Disease*.

[30] Gerhardt T., Rings D., Höblinger A., Heller J., Sauerbruch T., Schepke M. (2010). Combination of bilateral metal stenting and trans-stent photodynamic therapy for palliative treatment of hilar cholangiocarcinoma. *Zeitschrift für Gastroenterologie*.

[31] Kadayifci A., Atar M., Forcione D. G., Casey B. W., Kelsey P. B., Brugge W. R. (2016). Radiofrequency ablation for the management of occluded biliary metal stents. *Endoscopy*.

